# Th cytokine profile in childhood-onset systemic lupus erythematosus

**DOI:** 10.1186/s12887-021-02659-3

**Published:** 2021-04-21

**Authors:** Wei Quan, Jingnan An, Gang Li, Guanghui Qian, Meifang Jin, Chenxi Feng, Si Li, Xiaozhong Li, Yunyun Xu, Xiaohan Hu

**Affiliations:** 1 Institute of Pediatrics, Children’s Hospital of Soochow University, 92 Zhongnan St, Suzhou, Jiangsu Province China; 2 Department of Nephrology and Immunology, Children’s Hospital of Soochow University, 303 Jingde St, Suzhou, Jiangsu Province China; 3 Institute of Blood and Marrow Transplantation, The First Affiliated Hospital of Soochow University, Suzhou, China; 4 Medical College of Soochow University, Suzhou, China

**Keywords:** Childhood onset, Systemic lupus erythematosus, Th cytokine

## Abstract

**Background:**

Childhood-onset systemic lupus erythematosus (cSLE) is a kind of chronic inflammatory disease characterized by a highly abnormal immune system. This study aimed to detect the serum levels of Th (T helper) cytokines (IL-2, IL-4, IL-5, IL-6, IL-9, IL-10, IL-13, IL-17A, IL-17F, IL-21, IL-22, IFN-γ and TNF-α) in cSLE and healthy controls, and then to elucidate their association with clinical manifestations, disease activity and laboratory parameters. In order to provide clues for early diagnosis and timely intervention treatment of cSLE patients.

**Methods:**

A total of 33 children with cSLE and 30 healthy children were enrolled in this study. Children in the cSLE group were classified into the inactive or active cSLE group according to their SLE disease activity index 2000 (SLEDAI-2 K) score. Th cytokine profiles in the peripheral blood were detected and analysed.

**Results:**

Levels of IL-2, IL-10 and IL-21 in the cSLE group were significantly higher than those in the healthy control group (*P* < 0.05, *P* < 0.01 and *P* < 0.01, respectively). Expression of IL-2, IL-10 and IL-21 in the active cSLE group was significantly higher than that in the healthy control group (*P* < 0.05, *P* < 0.01 and *P* < 0.05, respectively), but that of IL-22 expression was markedly lower in the active cSLE group than in the healthy control group (*P* < 0.001). IL-21 in the inactive SLE group was significantly higher than that in the healthy control group (*P* < 0.05), and levels of IL-2 and IL-10 in the active cSLE group were significantly higher than those in the inactive cSLE group (*P* < 0.01 and *P* < 0.05). In-depth analysis showed that after excluding age, gender and drug interference, the levels of IL-2 (*P* < 0.05), IL-6 (*P* < 0.05) and IL-10 (*P* < 0.05) were still positively correlated with SLEDAI-2 K scores. However, the levels of IL-6 (*P* < 0.05) and IFN- γ (*P* < 0.05) were still negatively correlated with CD4^+^/CD8^+^, and the concentration of IL-6 (*P* < 0.05) was still positively correlated with the occurrence of nephritis.

**Conclusion:**

This study provides a theoretical basis for the discovery of effective methods to regulate imbalance in T lymphocyte subsets in cSLE, which may lead to new approaches for the diagnosis of cSLE.

## Background

Systemic lupus erythematosus (SLE) is an autoimmune disease caused by external environmental factors that act on the genetic susceptibility of an individual, stimulating the body’s immune regulation barriers and breaking the individual’s immune tolerance; this systemic autoimmune disease thus involves multiple systems and organs and shows high levels of clinical heterogeneity [1, 2]. Childhood-onset SLE (cSLE) accounts for 10–20% of SLE cases. In general, cSLE has become an extremely concerning disease due to its rapid development, increased likelihood of vital organ involvement, more severe clinical symptoms than adult SLE, poor prognosis, and high mortality [3].

cSLE, a chronic inflammatory disease caused by a highly abnormal immune system (including T cells, B cells and mononuclear macrophages), is characterized by the production of autoantibodies and the deposition of immune complexes [4]. cSLE, one of the most common autoimmune diseases in children, is immunologically characterized by strong proliferation, increased immunoglobulin, production of various autoantibodies and weak intracellular and extracellular immune responses [5, 6]. Although, the pathogenesis of this disease has not been elucidated to date, a large number of cytokines, signalling molecules and pattern recognition receptors in the immune system are involved [7]. Pro-inflammatory cytokines, such as those involved in local inflammatory responses that cause tissue damage, are involved in the immune disorders common in SLE patients [8]. Due to their key role in the pathogenesis of SLE, cytokines have become the focus of an increasing number of studies. Th cell subsets and cytokines were recently reported to play an important role in the pathogenesis of SLE [9].

Because of the higher probability that each organ is involved, cSLE is often more serious than SLE in adults; the disease process is also more dangerous than that in adults [10, 11]. Therefore, detection of cytokines in SLE patients is helpful for comprehensively understanding the immune status of the body and correctly judging the disease condition. However, studies involving the detection of Th cytokines have mostly been carried out in adult SLE, whereas similar studies in cSLE are rare and incomplete.

Accordingly, this study aimed to detect Th cytokine levels in cSLE and to explore their relationship with clinical manifestations, disease activity and laboratory factors, which may further provide a new idea for early diagnosis and timely intervention of cSLE.

## Methods

### General information

Patients with cSLE (*n* = 33; 6 males and 27 females) admitted to the Children’s Hospital affiliated with Soochow University from July 2018 to October 2018 were selected as the cSLE group. All patients met the 1997 American College of Rheumatology (ACR) revised SLE classification criteria [12]: nephritis (diagnosed based on 24-h urinary protein> 0.5 g), haematological manifestations (including leukopenia diagnosed by two or more tests less than 4000/mm^3^), and thrombocytopenia (fewer than 100 × 10^9^/L thrombocytes), excluding haemolytic anaemia caused by drug factors and other reasons, were diagnosed. Additional characteristic clinical manifestations of SLE such as malar erythema, skin mucosal lesions and arthritis were also evaluated. The cSLE cases were then scored using SLEDAI-2 K [13] developed at the University of Toronto, Canada. Children with an SLEDAI-2 K score ≤ 4 over the past 10 days were classified into the inactive group (inactive cSLE). Children with an SLEDAI-2 K score > 4 over the past 10 days were classified into the active group (active cSLE). Patients with other comorbid autoimmune diseases and inflammatory diseases were excluded. Healthy volunteers who underwent outpatient physical examination (*n* = 30; 5 males and 25 females) were selected as the healthy control group. This study was approved by the Ethics Committee of the Children's Hospital of Soochow University.

### Main experimental reagents and instruments

A LEGENDplex Human Th Cytokine Mix and Match Subpanel (BioLegend, USA) and flow cytometry analyser (Beckman Coulter, USA) were used. The minimum detection concentrations for each cytokine were as follows: IL-2, 4.44 pg/ml; IL-4, 5.02 pg/ml; IL-5, 3.84 pg/ml; IL-6, 4.07 pg/ml; IL-9, 4.22 pg/ml; IL-10, 2.73 pg/ml; IL-13, 3.88 pg/ml; IL-17A, 1.97 pg/ml; IL-17F, 4.35 pg/ml; IL-21, 3.80 pg/ml; IL-22, 6.93 pg/ml; IFN-γ, 7.47 pg/ml; TNF-α, 6.63 pg/ml.

### Detection of serum Th cytokines

Blood samples were collected from all subjects: 5 mL of peripheral venous blood was collected by physical examiners in the morning and allowed at room temperature for 2 h to separate the serum. The supernatant was centrifuged at 500 r/min for 10 min, after which the upper serum was collected and then stored in an Eppendorf tube at − 80 °C. Using thawed samples, the serum contents of IL-2, IL-4, IL-5, IL-6, IL-9, IL-10, IL-13, IL-17A, IL-17F, IL-21, IL-22, IFN-γ and TNF-α in each specimen were detected by cytometric bead array (CBA), namely, cytokine microsphere detection technology, and the results were recorded.

### Observation of clinical symptoms and relevant laboratory indicators of SLE patients

The clinical symptoms and data for all SLE children were recorded, including the following: their mental state; the presence of joint; skin and other related clinical symptoms; and history of medication use over the past 3 months. Laboratory indicators, anti-double-stranded DNA (anti-dsDNA) antibody, complement 3 (C3), and complement 4 (C4) were all measured by the Laboratory Department of the Children's Hospital of Soochow University.

### Statistical analyses

Raw data were analysed using LEGEND plex software from BioLegend (ver. 8.0), and the results are represented in units of pg/ml. Statistical analyses were performed using SPSS 21.0 (SPSS, Chicago, IL, USA). Normally distributed variables were expressed as the mean ± standard deviation (SD) and were compared by t-test. Nonnormally distributed variables were compared by Mann-Whitney U-test, and the results are expressed as the median (interquartile range). Categorical data were described as numbers (percentages). Differences between groups were assessed by using Fisher’s exact test. Correlations between different variables were analysed by Spearman correlation analysis. The data for continuous variables were log-transformed to satisfy the assumption of the homogeneity of variance. The multicollinearity was assessed by tolerance values and variance inflation factor (VIF), when tolerance> 0.1 or VIF < 10 indicated absence of multicollinearity. A *p* value< 0.05 indicated statistical significance.

## Results

### Demographics

We enrolled 33 children with SLE as the cSLE group; the group included 6 males and 27 females 2–19 years of age with a median age of 13 years. The shortest course of disease was the initial diagnosis; the longest was over 7 years. The mean age of onset was 11.21 ± 2.52 years, and the median course of disease was 6 months. The age of the individuals in the control group ranged from 5 to 18 years old, with a median age of 13 years. The cSLE group was divided into an active group (active cSLE) and an inactive group (inactive cSLE). Sixteen children aged 11–17 years, had inactive cSLE (SLEDAI-2 K score ≤ 4). Among these children, the average age of onset was 10.56 ± 3.08 years, and the median course of disease was 30 months. Seventeen children 9–17 years old, had active SLE (SLEDAI-2 K score > 4), with an average age of onset of 11.8 ± 1.74 years and a median course of disease of 2 months. Among the 30 healthy children who comprised the control group, 5 were male and 25 female; ages ranged from 6 to 17 years, and the median age was 13 years. There was no statistically significant difference (*P* > 0.05) in sex or age between the groups, and these values were comparable (Table 1).

**Table 1:** Characteristics of each study group

Variable	cSLE (*n* = 33)	Active cSLE (*n* = 17)	Inactive cSLE (*n* = 16)	Control (*n* = 30)	P_ad_	P_bd_	P_cd_	P_bc_
**Demographics**								
Female sex (%)	27 (81.82)	16 (94.12)	11 (68.75)	25 (83.33)	1.000	0.396	0.283	0.085
Age (years)	13.00 (11.00–15.00)	13.00 (12.00–14.00)	12.50 (11.00–17.00)	13.00 (11.00–14.00)	0.293	0.367	0.358	0.870
Age at disease onset (years)	11.21 ± 2.52	11.82 ± 1.74	10.56 ± 3.08	NA				
Disease duration (months)	6.00 (1.25–36.00)	2.00 (0.00–30.00)	30.00 (3.50–72.00)	NA				
SLEDAI-2 K score	8.48 ± 5.85	13.53 ± 3.38	3.13 ± 1.20	NA				
**Clinical manifestations**								
Malar erythema (%)	19 (57.58)	14 (82.35)	5 (31.25)	NA				
Nephritis (%)	25 (75.76)	13 (76.47)	12 (75.00)	NA				
Mucocutaneous disorder (%)	2 (6.06)	2 (11.76)	0 (0.00)	NA				
Arthritis (%)	3 (9.09)	3 (17.65)	0 (0.00)	NA				
Haematological disorder (%)	6 (18.18)	4 (23.53)	2 (12.50)	NA				
**Laboratory features**								
Anti-dsDNA (%)	15 (45.45)	11 (64.71)	4 (25.00)	NA				
Low C3 (%)	18 (54.55)	14 (82.35)	4 (25.00)	NA				
Low C4 (%)	16 (48.48)	10 (58.82)	6 (37.50)	NA				
CD4^+^/CD8^+^ inversion	33 (69.70)	14 (82.35)	9 (56.25)	NA				
**Medications**								
Prednisone (%)	27 (81.82)	15 (88.24)	12 (75.00)	NA				
Hydroxychloroquine (%)	23 (69.70)	10 (58.82)	13 (81.25)	NA				
Cyclophosphamide (%)	13 (39.39)	10 (58.82)	2 (12.50)	NA				
Mycophenolate mofetil (%)	16 (48.48)	11 (64.71)	5 (31.25)	NA				
Methotrexate (%)	1 (3.03)	1 (5.88)	0 (0.00)	NA				

*SLEDAI-2 K* systemic lupus erythematosus disease activity index 2000, *anti-dsDNA* anti-double-stranded DNA, *C3* complement 3, *C4* complement 4, *NA* not applicable, *P*_*ad*_ the cSLE group compared with the control group, *P*_*bd*_ the active cSLE group compared with the control group, *P*_*cd*_ the inactive cSLE group compared with the control group, *P*_*bc*_ the active cSLE group compared with the inactive cSLE group; values are expressed as the mean ± SD, median (interquartile range) or number (percentage)

### Clinical manifestations

For all cSLE, the main clinical manifestations were malar erythema (57.58%), nephritis (75.76%), haematological disorder (18.18%), mucocutaneous disorder (6.06%) and arthritis (9.09%). The incidence rates of the above clinical symptoms were 82.35, 76.47, 23.53, 11.76 and 17.65%, respectively, in active cSLE and 31.25, 75.00, 12.50, 0 and 0%, respectively, in inactive cSLE (Table 1).

### Laboratory parameters and treatments

The overall anti-dsDNA antibody positivity rate in the children with cSLE was 45.45%, and the incidence of low complement C3 and C4 was 54.55 and 48.48%, respectively. Among these parameters, anti-dsDNA antibody positivity rates in the active and inactive groups were 64.71 and 25.00%, respectively; the incidence of low complement C3 was 82 and 25%, respectively, and that of low complement C4 was 58 and 37%, respectively (Table 1).

In the cSLE group, 27 children (81.82%) received prednisone therapy, including 15 (88.24%) in the active stage and 12 (75%) in the inactive stage; 23 (41%) were treated with hydroxychloroquine, including 10 (58.82%) in the active stage and 13 (81.25%) in the inactive stage; 13 (39.39%) received cyclophosphamide, among which 10 (58.52%) in the active stage and 2 (12.50%) in the inactive stage; 16 (48.48%) were treated with mycophenolate mofetil among which 11 (64.71%) in the active stage and 5 (31.25%) in the inactive stage. Only 1 (5.88%) with active cSLE received methotrexate therapy (Table 1).

### Th cytokine profiles of each group

**Fig. 1:**
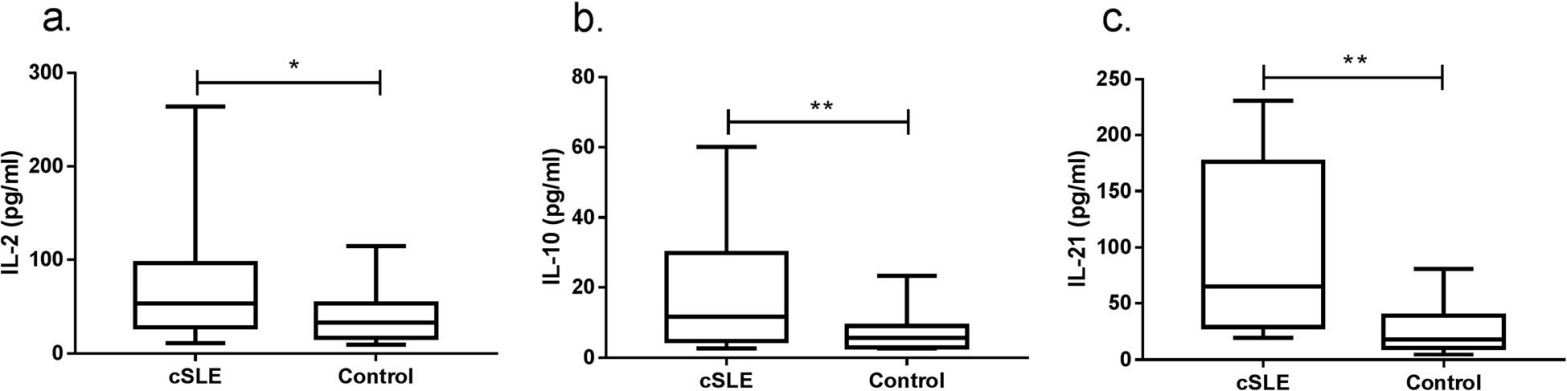
Differences in serum Th cytokine levels between the cSLE and control groups. **a**. IL-2; **b**. IL-10; **c**. IL-21. Mann-Whitney U-test; * *P* < 0.05; ** *P* < 0.01

**Fig. 2:**
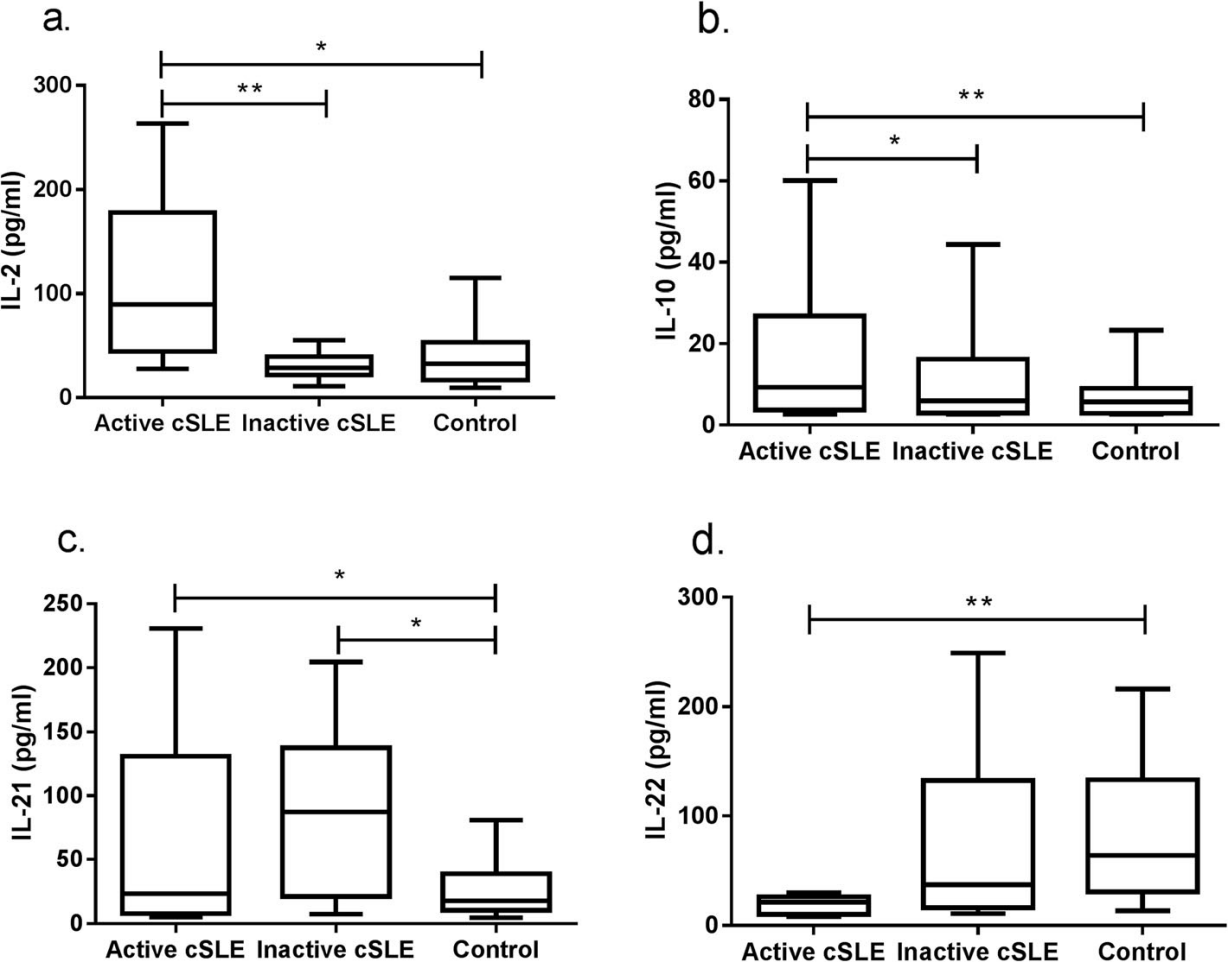
Differences in serum Th cytokines between the active cSLE, inactive cSLE and control groups. **a**. IL-2; **b**. IL-10; **c**. IL-21; **d**. IL-22. Mann-Whitney U-test; * *P* < 0.05; **P < 0.01

Serum levels of Th cytokines (IL-2, IL-4, IL-5, IL-6, IL-9, IL-10, IL-13, IL-17A, IL-17F, IL-21, IL-22, IFN-γ and TNF-α) in the cSLE group and healthy control group were quantified by the CBA method (Table 2, Figs. 1 and 2). Levels of IL-2, IL-10 and IL-21 in the cSLE group were significantly higher than those in the healthy control group (*P* < 0.05, *P* < 0.01 and *P* < 0.01, respectively) (Fig. 1a, b, c). Specifically, levels of IL-2, IL-10, and IL-21 were significantly higher in the active cSLE group than in the healthy control group (*P* < 0.05, *P* < 0.01 and *P* < 0.05, respectively), and levels of IL-22 were significantly lower in the active cSLE group than in the healthy control group (*P* < 0.001). The level of IL-21 in the inactive SLE group was significantly higher than that in the healthy control group, and the difference was statistically significant (*P* < 0.05). In addition, levels of IL-2 and IL-10 in the active cSLE group were significantly higher than those in the inactive cSLE group, a difference that was also significant (*P* < 0.01 and *P* < 0.05, respectively) (Fig. 2a, b, c, d).

**Table 2:** Comparison of Th cytokine concentrations in the different groups

Cytokine	cSLE (*n* = 33)	Active cSLE (*n* = 17)	Inactive cSLE (*n* = 16)	Control (*n* = 30)	P_ad_	P_bd_	P_cd_	P_bc_
IL-2	53.11 (27.80–96.71)	89.99 (59.49–178.10)	28.80 (21.64–39.34)	32.51 (16.90–53.39)	0.047*	0.012*	0.395	0.003**
IL-4	31.73 (12.20–76.14)	14.17 (10.88–45.61)	51.08 (15.40–126.39)	41.28 (31.39–80.06)	0.565	0.143	0.806	0.162
IL-5	5.91 (4.36–11.89)	8.24 (4.92–13.91)	5.07 (4.54–9.34)	6.47 (4.83–9.53)	0.802	0.537	0.783	0.619
IL-6	41.80 (19.03–126.67)	41.80 (18.73–87.16)	70.02 (17.72–139.57)	36.00 (18.63–61.98)	0.245	0.438	0.302	0.490
IL-9	43.24 (23.52–64.37)	46.23 (18.00–112.15)	43.24 (26.45–62.75)	35.82 (16.38–76.85)	0.438	0.482	0.912	0.452
IL-10	11.54 (4.80–29.82)	13.30 (9.98–33.67)	5.94 (2.97–16.25)	5.71 (2.91–9.03)	0.001**	0.001**	0.162	0.039*
IL-13	18.29 (8.57–47.65)	18.20 (7.30–40.57)	18.76 (8.81–56.03)	26.81 (16.22–46.22)	0.253	0.138	0.739	0.423
IL-17A	38.90 (12.18–453.90)	94.56 (24.29–450.40)	16.25 (11.55–458.13)	45.42 (17.92–79.22)	0.291	0.522	0.483	0.937
IL-17F	17.30 (5.37–22.44)	11.34 (4.95–21.34)	20.52 (12.12–59.75)	19.19 (9.95–44.56)	0.366	0.235	0.906	0.249
IL-21	65.33 (29.17–176.17)	55.07 (27.55–183.42)	87.18 (29.17–137.60)	18.01 (10.51–39.17)	0.003**	0.047*	0.044*	0.807
IL-22	24.11 (13.35–63.48)	21.43 (10.02–26.28)	37.34 (16.43–132.33)	63.85 (30.84–133.13)	0.217	< 0.001**	0.972	0.086
IFN-γ	72.95 (16.59–227.02)	58.80 (18.17–336.42)	72.95 (8.96–205.93)	69.61 (30.04–148.88)	0.555	0.524	0.883	0.652
TNF-α	46.95 (21.35–114.53)	108 (25.29–131.23)	25.03 (13.48–77.26)	17.50 (11.68–30.65)	0.151	0.150	0.297	0.877

*IL-2* Interleukin-2, *IL-4* Interleukin-4, *IL-5* Interleukin-5, *IL-6* Interleukin-6, *IL-9* Interleukin-9, *IL-10* Interleukin-10, *IL-13* Interleukin-13, *IL-17A* Interleukin-17A, *IL-17F* Interleukin-17F, *IL-21* Interleukin-21, *IL-22* Interleukin-22, *IFN-γ* Interferon-γ, *TNF-a* Tumour necrosis factor-α, *P*_*ad*_ the cSLE group compared with the control group, *P*_*bd*_ the active cSLE group compared with the control group, *P*_*cd*_ the inactive cSLE group compared with the control group, *P*_*bc*_ the active cSLE group compared with the inactive cSLE group; *P < 0.05, **P < 0.01

### Correlation analysis of Th cytokine levels with SLEDAI-2 K score, CD4^+^/CD8^+^ and nephritis

The Spearman linear correlation test was applied to analyse the correlation between the SLEDAI-2 K score and levels of different cytokines. Levels of IL-2 (r = 0.382, *P* = 0.028), IL-6 (r = 0.514, *P* = 0.002) and IL-10 (r = 0.429, *P* = 0.016) correlated positively with the SLEDAI-2 K score (Table 3). In contrast, levels of other Th cytokine did not correlate significantly with the SLEDAI-2 K score. For the cSLE, no significant association between different cytokine levels and drug therapies was found. We further conducted multiple linear regression analysis, and after excluding the interference of confounding factors such as age, gender and medications on cytokine levels, it was found that there was still a linear relationship between IL-2 (*P* < 0.05), IL-6 (*P* < 0.05), IL-10 (*P* < 0.05) and SLEDAI-2 K, respectively (Table 4). The correlation analysis between CD4^+^/CD8^+^ and different levels of cytokines showed that the levels of IL-6 (r = − 0.381, *P* = 0.029), IL-17A (r = − 0.375, *P* = 0.041) and IFN-γ (r = − 0.445, *P* = 0.011) were negatively correlated with CD4^+^/CD8^+^ (Table 3). Further multiple linear regression analysis showed that IL-6 (*P *< 0.05) and IFN- γ (*P* < 0.05) still had a linear relationship with CD4^+^/CD8^+^ after excluding the interference of age, gender and drugs (Table 4). The correlation analysis between nephritis and different concentrations of cytokines showed that the levels of IL-6 (r = 0.368, *P* = 0.035) and TNF-α (r = 0.415, *P* = 0.031) were positively correlated with the occurrence of nephritis (Table 3). Multiple linear regression analysis showed that there was still a linear relationship between IL-6 (*P* < 0.05) and nephritis after excluding age, gender and drug interference (Table 4).

**Table 3:** Correlation between different variables and cytokine levels in cSLE

Cytokine	SLEDAI-2 K score		CD4^+^/CD8^+^		Nephritis	
	r	*P* value	r	*P* value	r	*P* value
IL-2	0.382	0.028*	0.028	0.887	−0.176	0.329
IL-4	0.188	0.348	−0.277	0.162	0.365	0.061
IL-5	0.173	0.387	−0.150	0.456	0.067	0.738
IL-6	0.514	0.002**	−0.381	0.029*	0.368	0.035*
IL-9	0.310	0.141	−0.270	0.201	0.264	0.212
IL-10	0.429	0.016*	−0.265	0.149	0.227	0.219
IL-13	0.104	0.565	−0.220	0.219	0.284	0.109
IL-17A	0.113	0.553	−0.375	0.041*	0.236	0.210
IL-17F	0.260	0.157	−0.256	0.164	0.252	0.172
IL-21	0.302	0.112	−0.030	0.876	0.328	0.083
IL-22	−0.024	0.893	0.027	0.886	0.298	0.109
IFN-γ	0.085	0.642	−0.445	0.011*	0.314	0.080
TNF-α	0.217	0.277	−0.106	0.599	0.415	0.031*

Spearman correlation analysis; *P < 0.05, **P < 0.01

**Table 4:** Clinical and laboratory factors potentially associated with different variables

Variables	B	SE	*P* value
**SLEDAI-2 K scores**			
IL-2	0.036	0.016	0.039*
gender	3.573	2.430	0.154
age	−0.116	0.372	0.757
Prednisone	1.934	3.278	0.561
Hydroxychloroquine	0.378	2.475	0.880
Cyclophosphamide	−2.161	2.490	0.394
Mycophenolate mofetil	−1.946	3.326	0.564
Methotrexate	−8.689	5.906	0.154
IL-6	0.050	0.019	0.016*
gender	4.097	2.376	0.097
age	−0.276	0.366	0.454
Prednisone	2.606	3.199	0.423
Hydroxychloroquine	0.724	2.415	0.767
Cyclophosphamide	−3.712	2.427	0.139
Mycophenolate mofetil	−0.825	3.217	0.800
Methotrexate	−9.794	5.738	0.101
IL-10	0.171	0.077	0.036*
gender	4.336	2.520	0.099
age	0.165	0.401	0.685
Prednisone	1.602	3.349	0.637
Hydroxychloroquine	2.318	2.898	0.432
Cyclophosphamide	−3.114	2.549	0.234
Mycophenolate mofetil	−2.926	3.542	0.417
Methotrexate	−7.625	6.033	0.219
**CD4**^**+**^**/CD8**^**+**^			
IL-6	−0.003	0.002	0.045*
gender	0.102	0.185	0.586
age	0.018	0.027	0.509
Prednisone	0.319	0.213	0.148
Hydroxychloroquine	−0.046	0.199	0.821
Cyclophosphamide	−0.257	0.192	0.193
Mycophenolate mofetil	−0.192	0.277	0.495
Methotrexate	0.279	0.429	0.521
IFN-γ	−0.002	0.001	0.025*
gender	−0.341	0.191	0.859
age	0.046	0.023	0.063
Prednisone	0.070	0.214	0.746
Hydroxychloroquine	0.061	0.179	0.738
Cyclophosphamide	−0.214	0.167	0.213
Mycophenolate mofetil	−0.022	0.237	0.927
Methotrexate	0.296	0.379	0.443
**Nephritis**			
IL-6	0.003	0.001	0.036*
gender	0.007	0.189	0.970
age	−0.056	0.029	0.064
Prednisone	0.156	0.237	0.516
Hydroxychloroquine	−0.054	0.206	0.795
Cyclophosphamide	−0.087	0.202	0.671
Mycophenolate mofetil	0.344	0.301	0.263
Methotrexate	−0.056	0.445	0.901

Continuous variables were log-transformed in the linear regression analyses, there was no multicollinearity among different variables

*SE* standard error; *P < 0.05

## Discussion

cSLE, a common chronic systemic autoimmune disease in children, involves multiple systems and organs throughout the body; its clinical manifestations are complex, the course of the disease is protracted, and the disease can recur [14]. The pathogenesis of cSLE is not fully clear, but it mainly involves dysregulation of the immune system, including excessive T and B cell activation, resulting in the production of large amounts of a variety of autoantibodies, and immune complex deposition, which causes multiple organ damage [4, 15]. Disruption of the immune state may be the key mechanism involved in a range of autoimmune diseases, including SLE. For example, T cell-mediated immunoreaction plays an important role in specific immunity. One of the characteristics of SLE is abnormalities in the differentiation and regulation of T cells. Previous studies found that the pathogenesis of SLE is related to an imbalance in the proportion of regulatory T (Treg) cells and Th cells and an increase in the proportion of Th cells [16]. The Th subgroup itself is also altered during the development of SLE. In recent years, Th cells have been found to have a key role in autoimmune diseases by secreting a variety of cytokines and mediating interactions between cells. Furthermore, overexpression of IL-6, IL-10, IL-17, and TNF-α, among others, is important in the pathogenesis of SLE, polymyositis (PM), dermatomyositis (DM) and rheumatoid arthritis (RA) and is significantly related to disease activity [17–19]. SLE is a prototypical autoimmune disease. Cytokines have crucial functions in the pathogenesis of SLE and determine the degree of disease activity.

In this study, serum IL-2 levels were found to be increased in cSLE and correlate positively with disease activity. Moreover, the level of IL-2 in active cSLE was significantly higher than that in inactive cSLE. Previous studies have shown that people deficient in IL-2 can develop severe autoimmune diseases, possibly due to the uncontrolled proliferation of autoreactive T cells and B cells and the proliferation of immature, non-functional Tregs caused by these defects [20]. Other researches revealed that disturbances in the Treg-IL-2 axis were associated with increased disease activity, which highlighting the importance of IL-2 deficiency in pathogenesis of adult SLE. While, the homeostasis and phenotypic abnormalities in the Treg population caused by IL-2 deficiency were associated with the development of lupus nephritis [21, 22]. Interestingly, based on laboratory test results in our study, serum IL-2 deficiency is not observed in cSLE, especially active SLE. Therefore, the role of IL-2 in the pathogenesis of cSLE remain to be clarified by further analysis.

IL-6, which can be secreted by a variety of immune cells, mainly functions to stimulate the proliferation and differentiation of B cells and their development into mature B cells capable of secreting antibodies. Our study showed that the expression level of serum IL-6 in cSLE correlated positively with the occurrence of lupus nephritis and degree of disease activity, and negatively with CD4^+^/CD8^+^, indicating a potential correlation between IL-6 and the pathogenesis of SLE. In studies of SLE in adults, IL-6 was found to be associated with anaemia in those with lupus nephritis. Furthermore, more severe anaemia was associated with a higher IL-6 concentration [23]. Other researches on adult SLE have shown that high level of IL-6 were associated with increased disease activity and ds-DNA titers [24, 25], meanwhile IL-6 was significantly elevated in the serum of patients with lupus nephritis [23], our study produced similar results. IL-6 can also stimulate activation of the STAT3 signalling pathway, reduce the cell circulation speed in the blood and prevent apoptosis in immune cells [26]. In addition, IL-6 can inhibit the proliferation of Tregs and promote the development of autoimmunity [27]. All these results suggest that IL-6 may serve as a predictor of SLE.

There is a balance disorder between CD4^+^T cells and CD8^+^T cells in patients with SLE, which leads to the inversion of the proportion of CD4^+^/CD8^+^T cells. The main manifestation of this change is the decrease of CD4^+^T cells level, accompanied by dysfunction of Th1 and Th2 cells, which leads to immune regulation disorder and abnormal cytokine secretion, the active function of B lymphocytes and the production of a large number of autoantibodies. At the same time, the hyperfunction of CD8^+^T cells participates in tissue injury, which further leads to immune disorder and aggravation of immunopathological damage [28, 29]. Our study showed that CD4^+^/CD8^+^ was negatively correlated with IFN-γ, that is, there was an increase in IFN-γ and an inversion of CD4^+^/CD8^+^ T cells in cSLE. IFN- γ is secreted by Th1 cells. Study have shown that the level of serum IFN- γ in patients with glomerulonephritis in SLE is significantly higher than that in patients without glomerulonephritis, which may be related to the fact that IFN- γ promotes the formation and deposition of immunoglobulin in the kidney [30], indicating that IFN- γ is involved in the immune disorder process of SLE.

In this study, the serum IL-10 level in cSLE was significantly higher than that in the healthy group, and the IL-10 level in the active stage was significantly higher than that in the inactive stage. IL-10 is an anti-inflammatory cytokine, and Th2 cells as well as various types of regulatory T cells are generally thought to be the source of its production in T cells. IL-10 stimulates B cell proliferation and IgG synthesis. Previous studies have confirmed that IL-10 is a susceptibility gene for SLE. In adult patients with SLE, serum levels of IL-10 correlate positively with the SLEDAI-2 K score and anti-dsDNA antibody levels, consistent with the results of the present study. IL-10 can inhibit cytokine production, downregulate monocyte antigen presentation and co-stimulation, and inhibit T cell proliferation, thereby increasing immunosuppression and achieving anti-inflammatory effects [31]. Although IL-10 plays a typical role in the immune process, recent studies have shown that IL-10 functions cytokine activation and induction, which are related to the pathogenesis of SLE. Therefore, the role of IL-10 in cSLE still needs more studies to clarify.

We found that serum IL-21 levels in cSLE were significantly higher than those in healthy controls, and IL-21 levels in the inactive cSLE group were markedly higher than those in the healthy control group. IL-21 can activate B cells to secrete IgG1 and IgG3 and induce all B cell subsets to differentiate into Ig-secreting cells, thus producing large amounts of IgM, IgG and IgA [32]. IL-21 produced by Follicular helper T (Tfh) cells plays a major role in the initial immune response, secondary immune response, and long-term maintenance of humoural immunity by B cells in response to T cell-dependent antigens. Terrier et al. found that serum IL-21 levels in adult SLE patients were significantly increased compared with those in a healthy control group [33], which is consistent with the results of our study. Thus, the significant increase in Tfh cell-related cytokine IL-21 levels in the serum of children with cSLE suggests that these cells also play a crucial role in the pathogenesis of cSLE. The immune system stimulates B cells by producing cytokines such as IL-21, causing abnormal humoural immune responses and participating in the pathogenesis of cSLE. In view of this, IL-21 may become a target molecule for the treatment of SLE in children, and this study of Tfh cell-related cytokines will also lead to new approaches for their treatment.

IL-22 is produced by a variety of cell types, including Th17 cells, natural killer (NK) cells, and Th22 cells. Th22 cells are the main cell type that secretes IL-22. Research by Lin et al. showed that in patients with newly diagnosed SLE, the concentration of IL-22 was reduced compared with that in patients with relapsed SLE and healthy controls [34]. In our study, the serum IL-22 concentration in cSLE was lower than that in the control group, and the IL-22 level in children with active disease was significantly different from that of children in the control group, consistent with the results of a previous study on SLE in adults. This finding suggests that IL-22 plays an opposite role in the pathogenesis of this disease. Nevertheless, in a study by Zhao et al., the serum IL-22 concentration in SLE patients was significantly higher than that in the healthy control group. After glucocorticoids were administered, the number of cells associated with IL-22 secretion was reduced. IL-22 is believed to play a role in the development of SLE. Under normal circumstances, Th22 cells, Th17 cells, Treg cells and other cell subsets interact and regulate each other to maintain a state of immune equilibrium. In chronic inflammatory diseases, loss of the functions of key transcription factors that regulate Th22 cell differentiation exacerbates the occurrence of chronic inflammatory diseases [35]. The decreased number of Th22 cells in SLE patients may be because AhR, a key transcription factor that regulates Th22 cell differentiation, directly or indirectly controls the production and secretion of inflammatory cytokines such as IL-22, leading to immune imbalance in different immune cell subsets [36, 37]. This also suggests that IL-22 levels may be used as a target and auxiliary marker for the diagnosis and treatment of cSLE.

Our study has several limitations. First, due to the minimum detection concentration in our detection kit, we eliminated data below the lower limit of detection, which may have interfered with the research results. Second, despite no correlation between different drugs and cytokine levels detected, the fact that patients were treated with drugs before the analysis may have influenced the results to some extent. Third, this was a small-sample study, and further randomized and controlled clinical trials are needed to determine the efficacy of these cytokines in predicting activity in cSLE. Regardless, the strength of our study was that for the first time, we comprehensively analysed the level of serum Th cytokines in cSLE and its correlation with disease activity.

## Conclusion

Our research results show significant immunomodulatory disorder and abnormal expression of various Th cytokines closely related to the clinical manifestations, results of laboratory examination and disease activity in children with cSLE. The cytokines IL-2, IL-6, IL-10, IL-21 and IL-22 are candidate biomarkers for predicting childhood cSLE activity and may be used as targets in immunotherapy.

## Data Availability

The datasets analysed during the current study are available from the corresponding author on reasonable request.

## Abbreviations

SLE: Systemic lupus erythematosus
cSLE: Childhood-onset systemic lupus erythematosus
SLEDAI-2 K: Systemic lupus erythematosus disease activity index 2000
dsDNA: Double-stranded DNA
VIF: Variance inflation factor
SE: Standard error
GC: Germinal center
ACR: American College of Rheumatology
CBA: Cytometric bead array
C3: Complement 3
C4: Complement 4
IL: Interleukin
IFN: Interferon
TNF: Tumour necrosis factor
Th: T helper
Tfh: Follicular helper T
Treg: Regulatory T
NK: Natural killer
PM: Polymyositis
DM: Dermatomyositis
RA: Rheumatoid arthritis
